# Cellular Specificity and Inter-cellular Coordination in the Brain Bioenergetic System: Implications for Aging and Neurodegeneration

**DOI:** 10.3389/fphys.2019.01531

**Published:** 2020-01-08

**Authors:** Guoyuan Qi, Yashi Mi, Fei Yin

**Affiliations:** ^1^Center for Innovation in Brain Science, University of Arizona Health Sciences, Tucson, AZ, United States; ^2^Department of Pharmacology, College of Medicine Tucson, Tucson, AZ, United States; ^3^Graduate Interdisciplinary Program in Neuroscience, University of Arizona, Tucson, AZ, United States

**Keywords:** mitochondria, neuron, astrocyte, microglia, metabolic shift, metabolic coupling, brain aging, neurodegenerative diseases

## Abstract

As an organ with a highly heterogenous cellular composition, the brain has a bioenergetic system that is more complex than peripheral tissues. Such complexities are not only due to the diverse bioenergetic phenotypes of a variety of cell types that differentially contribute to the metabolic profile of the brain, but also originate from the bidirectional metabolic communications and coupling across cell types. While brain energy metabolism and mitochondrial function have been extensively investigated in aging and age-associated neurodegenerative disorders, the role of various cell types and their inter-cellular communications in regulating brain metabolic and synaptic functions remains elusive. In this review, we summarize recent advances in differentiating bioenergetic phenotypes of neurons, astrocytes, and microglia in the context of their functional specificity, and their metabolic shifts upon aging and pathological conditions. Moreover, the metabolic coordination between the two most abundant cell populations in brain, neurons and astrocytes, is discussed regarding how they jointly establish a dynamic and responsive system to maintain brain bioenergetic homeostasis and to combat against threats such as oxidative stress, lipid toxicity, and neuroinflammation. Elucidating the mechanisms by which brain cells with distinctive bioenergetic phenotypes individually and collectively shape the bioenergetic system of the brain will provide rationale for spatiotemporally precise interventions to sustain a metabolic equilibrium that is resilient against synaptic dysfunction in aging and neurodegeneration.

## Introduction

Active metabolism is fundamental in maintaining the life and activity of organisms by mediating the exchange of material, energy, and information with the environment. Most organisms rely on the catabolism of organic molecules to obtain energy. The acquirement of mitochondria by eukaryotic cells *via* endosymbiosis is proposed to remarkably expand their genomic capacity, which energetically drives and enables the evolution from single- to multi-cell systems (Lane and Martin, 2010; Lane, 2014). In higher organisms like the mammals, the production, storage, and utilization of fuels have been compartmentalized to, and distributed among, different organs. Organs such as liver, muscle, heart, and adipose tissue have adapted to possess specialized metabolic capacity and flexibility in using different fuels to meet their diverse functional needs.

Among all organs, brain is unique, not only because it consumes 20% of total glucose with its 2% of body weight (Bélanger et al., 2011), but also due to its unique metabolic profile and metabolite pool that are separated from the rest of the body by the blood–brain barrier (BBB). Although brain ultimately uses substrates from the periphery, its capacity to uptake and metabolize them varies from one fuel to another, with glucose being the predominant preference (Lundgaard et al., 2015). Within the brain, the highly heterogeneous cellular composition comes with diverse energetic capacity and fuel preference across cell types. While previous studies have established the vital role of bioenergetics system in brain aging and neurodegenerative diseases, the physiological and pathological roles of different cell types and their metabolic coordination are still being actively investigated. Accordingly, most strategies to restore brain bioenergetic homeostasis in neurodegenerative diseases either indistinguishably targeted all cell types or focused on neurons with limited consideration of the bioenergetic contributions from non-neuronal cells. In this review, we sought to discuss recent advances in revealing cell-type-specific bioenergetic role of brain cells with connection to their specialized functions. Moreover, inter-cellular metabolic coordination between neurons and astrocytes is reviewed in the context of brain aging and age-associated neurodegenerative disorders.

## Diverse Metabolic Phenotypes of Brain Cells

The mammalian brain is composed of diverse, specialized cell populations, including neurons, astrocytes, oligodendrocytes, microglia, and others. All of them together not only enable the highly refined electrophysiological activities, but also fulfill the organ’s nutritional needs and its defense against pathogens (Saunders et al., 2018). Recently, high-throughput single-cell RNA sequencing has achieved an unprecedented resolution in distinguishing and clustering cell types and sub-cell types in both rodent and human brains (Zeisel et al., 2015; Lake et al., 2016; Saunders et al., 2018). Moreover, studies in aging and degenerating brains also suggested that bioenergetic genes in different cell types are differentially altered in aging or diseases. Comparative analysis of bulk tissue- and single-cell RNA-seq of Alzheimer’s brains revealed distinct transcriptional changes across cell types, and a decline in mitochondrial genes at tissue level is only seen in neurons but not in glial cells from the same region of brain (Mathys et al., 2019).

**Figure 1:**
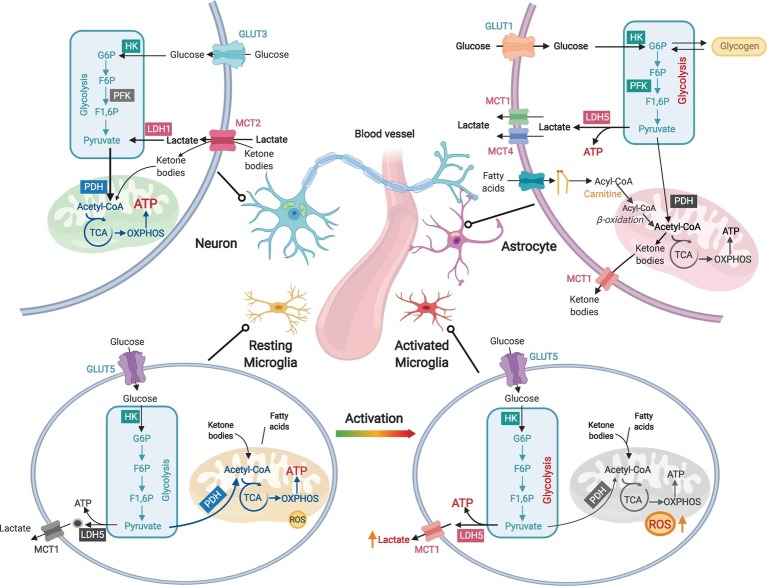
Diverse metabolic phenotypes of brain cells. Neurons produce ATP primarily *via* mitochondrial oxidative phosphorylation (OXPHOS), but their glycolytic rate is less active than that of astrocytes due to suppressed PFK activity. Both lactate and ketone bodies can enter neurons *via* monocarboxylate transporter 2 (MCT2). Astrocytes are highly active in glycolysis to produce lactate but less dependent on OXPHOS due to low pyruvate dehydrogenase (PDH) activity. Lactate is generated by lactate dehydrogenase 5 (LDH5) in astrocytes and exported by MCT1 and MCT4. Astrocytes are capable to metabolize fatty acids through β-oxidation. Fatty acids transport into mitochondria is facilitated by carnitine and carnitine palmitoyltransferases (CPTs). Ketone bodies can be generated in astrocytic mitochondria *via* ketogenesis. Excessive astrocytic glucose can be stored as glycogen, which can be converted back to glucose. Resting microglia have high rate of OXPHOS and low production of reactive oxygen species (ROS), whereas activated microglia produce large amount of ROS and undergo a metabolic reprogramming from OXPHOS toward glycolysis and lactate production.

Consistent with their transcriptomic profiles, different brain cell types have their unique metabolic phenotypes (Magistretti and Allaman, 2015; Figure 1). Both glia and neurons have the capacity to fully metabolize glucose, but their functional diversity differentiates their metabolic preference. Neurons, which generate and consume most brain ATPs, primarily rely on mitochondrial oxidative phosphorylation (OXPHOS) for energy transduction and possess low glycolytic capacity due to the suppression of a key glycolysis enzyme 6-phosphofructo-2-kinase/fructose-2,6-bisphosphatase (PFKFB) (Schönfeld and Reiser, 2013; Zheng et al., 2016; Figure 1). For non-glucose substrates, neurons have a low capacity to utilize fatty acids (Schönfeld and Reiser, 2013) but can metabolize ketone bodies generated in non-neuronal cells from fatty acid β-oxidation.

As the most abundant cell type in the brain, astrocytes provide critical metabolic and structural support to neurons, including modulation of ion homeostasis, supply of nutrients, and control of BBB permeability (Suzuki et al., 2011; Bolaños, 2016; Boison and Steinhäuser, 2018). In contrast to neurons, astrocytes maintain lower OXPHOS activity but higher glycolysis rate (Bélanger et al., 2011; Bouzier-Sore and Pellerin, 2013; Figure 1). Large amount of glucose metabolized by glycolysis in astrocytes is released as lactate, which serves as important metabolic fuel for neurons (Bolaños, 2016). Astrocytes also store excessive fuels in glycogen that can rapidly regenerate glucose for glycolysis or glutamate synthesis (Suzuki et al., 2011). Multiple lines of evidence suggest that astrocytes can metabolize non-glucose substrates including fatty acids (Ioannou et al., 2019), glutamate (Schousboe et al., 2014), and ketone bodies (McNair et al., 2017).

Microglia are resident immune cells in the brain (Viader et al., 2015), and are closely related to host defense against pathogens and CNS disorders (Perry et al., 2010). The survival and activation of microglia depend on sufficient energy supply. Cell-type-specific RNA-seq analysis reveals that microglia express the full set of genes required for both glycolysis and OXPHOS (Zhang et al., 2014), but their bioenergetic phenotype is activation-state-dependent (Aldana, 2019). Resting microglia depend mainly on OXPHOS for ATP production, whereas activated microglia favor glycolysis as manifested by increased lactate production and decreased mitochondrial oxygen consumption (Ghosh et al., 2018; Aldana, 2019; Figure 1). Additionally, transcriptome data suggest that microglia express key enzymes for fatty acids mobilization and β-oxidation, which may alternatively meet their elevated energy demand upon activation (Zhang et al., 2014).

A byproduct of mitochondrial OXPHOS is the electron leak from respiratory chain to generate superoxide and other reactive oxygen species (ROS; Murphy, 2009). Increased ROS production and oxidized redox status characterizes brain aging and neurodegeneration (Markesbery, 1997; Cadenas and Davies, 2000; Lin and Beal, 2006; Yin et al., 2012a, 2014; Hwang, 2013). Of all brain cell types, neurons are particularly susceptible to redox changes due to their high metabolic rate and limited antioxidant capacity (Baxter and Hardingham, 2016). Astrocytes, in contrast, have greater antioxidative potential (Gupta et al., 2012). Multiple studies have demonstrated that the astrocytic support of neuronal antioxidant system is a key neuroprotective mechanism against oxidative damage (Magistretti and Allaman, 2013). Since the electrons used to reduce ROS are ultimately from NADPH, which is generated by the pentose phosphate pathways (PPP) from glucose or by mitochondrial enzymes including the transhydrogenase (Yin et al., 2012b), elevated oxidative stress could cause a switch in re-routing fuels toward NADPH production rather than ATP production (Agarwal et al., 2013). Accordingly, astrocytic oxidative stress dampens glucose uptake and diverts glucose into PPP for NADPH production, which subsequently decreases lactate release from astrocytes to neurons and compromises the neuronal redox homeostasis (Steele and Robinson, 2012). The low capacity to metabolize fatty acids of neurons is another cause for its sensitivity to oxidative challenges: a recent study revealed that excess ROS trigger lipids synthesis in neuron and give rise to neurodegeneration if managed improperly (Liu et al., 2017).

## Intra-Cellular Bioenergetic Shifts

Glucose is brain’s dominating fuel under physiological conditions. Upon limited glucose availability, the brain undergoes a metabolic shift to use ketone bodies – including acetate, acetoacetate, and β-hydroxybutyrate – as its alternative energy source (Cunnane et al., 2011). Upon fasting, starvation, extended exercise, pregnancy, or development, fatty acids are mobilized from adipocytes and transported to liver for conversion to ketone bodies, which are subsequently transported to the brain to generate acetyl-CoA and eventually ATP in mitochondria (Drenick et al., 1972; Puchalska and Crawford, 2017; Mattson et al., 2018). Ketogenic interventions were reported with beneficial effect on cases of inborn metabolic disorder involving genetic deficiency of GLUT1 or pyruvate dehydrogenase, suggesting ketones as brain fuels to bypass glucose hypometabolism (Falk et al., 1976; Klepper, 2004; Wang et al., 2005). Although liver is believed to be the predominant supplier of ketone bodies, it was found that ketone bodies could also be generated in astrocytes from fatty acids (Martinez-Outschoorn et al., 2012). In brain aging and a variety of neurodegenerative diseases, a decline in glucose metabolism has been well-established (Ott et al., 1999; Fabre et al., 2002; Cunnane et al., 2011; Ding et al., 2013; Goyal et al., 2017). Under this situation, the brain may adapt a similar bioenergetic shift from exclusively relying on glucose toward using ketone bodies in response to energetic deficits (Ding et al., 2013; Klosinski et al., 2015; Camandola and Mattson, 2017). Beneficial effect of ketogenic diet on cognitive and cardiovascular outcomes has been observed in aging mice (Newman et al., 2017) and patients with mild cognitive impairment, a transition state toward AD (Krikorian et al., 2012).

While mitochondrial function in neurons is known to be impaired during aging, astrocytes exhibit elevated mitochondrial respiration in aging rat brains (Jiang and Cadenas, 2014), which is consistent with human studies showing increased astrocytic TCA activity with age (Boumezbeur et al., 2010). In parallel with these bioenergetic changes, rodent and human studies also demonstrated that the expression of glial fibrillary acidic protein (GFAP), a marker protein for activated astrocytes, increases with age (Nichols et al., 1993; Wu et al., 2005). Overall, age-associated activation of astrocytic OXPHOS could restrict their capacity to supply neurons with substrates such as lactate or glucose (Yan et al., 2013; Rodríguez-Arellano et al., 2016), and is connected with a switch from being neurotrophic to neurotoxic and to neuroinflammation (Jiang and Cadenas, 2014).

Similar to their peripheral counterparts macrophages, the bioenergetics of microglia also undergo a switch from OXPHOS toward glycolysis upon activation (Orihuela et al., 2016). LPS-activation of microglia-like BV-2 cell blocks mitochondrial oxygen consumption while activating anaerobic glycolysis and PPP (Voloboueva et al., 2013; Gimeno-Bayón et al., 2014). Consistently, glucose transporters GLUT1 and GLUT4, and hexokinase 2 (HK2), are upregulated in activated microglia (Gimeno-Bayón et al., 2014). Such a metabolic switch is critical to enable the fast response of activated microglia to energy-demanding processes such as proliferation, migration, cytokine secretion, and phagocytosis, because of the much faster rate in generating ATP by glycolysis than OXPHOS (Orihuela et al., 2016). Activation of microglia and chronic neuroinflammation are key features of aging and neurodegeneration. Upon LPS challenge, microglia from aged animals produce more ROS, compared to those isolated from young mouse brains (Tichauer et al., 2014; Figure 1). In a familial AD mouse model, exposure to amyloid-β triggers acute microglial activation in parallel with a metabolic shift from OXPHOS to glycolysis (Baik et al., 2019). A similar shift was also found in microglia from multiple sclerosis patients (Van Der Poel et al., 2019).

## Inter-Cellular Metabolic Communications

### Astrocyte-Neuron Lactate Shuttle

**Figure 2:**
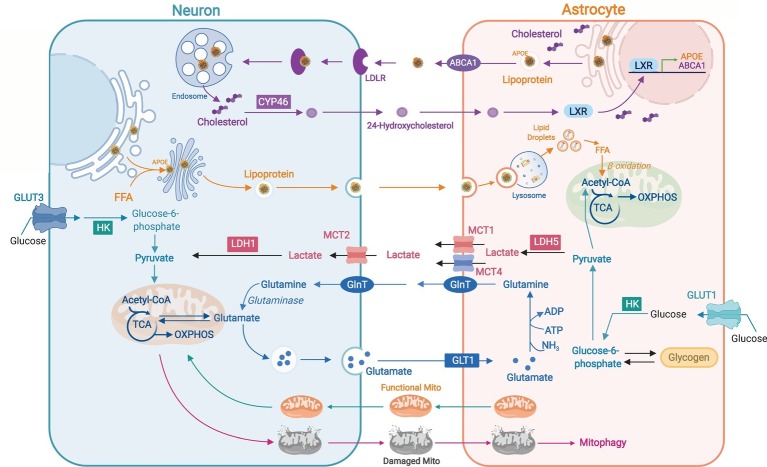
Neuron-astrocyte metabolic coordination. Astrocyte-synthesized cholesterols are packaged in APOE-associated lipoproteins and exported by ATP-binding cassette transporter A1 (ABCA1); released lipoproteins bind to neuronal LDL receptors and are internalized for neuronal use or conversion to 24-hydroxycholesterol (24-OHC) by cholesterol 24-hydroxylase (CYP46). 24-OHC produced by neurons can activate astrocytic live X receptor (LXR) to induce the expression of APOE and ABCA1. Hyperactive neurons release excessive free fatty acids (FFAs) in APOE-associated lipid particles to astrocytes where FFAs are targeted to lipid droplets for subsequent degradation by mitochondrial β-oxidation. High glycolytic rate in astrocytes produces lactate that can be transported to neurons by MCTs and used for ATP production. Neurotransmitter glutamate released by neurons is uptaken by astrocytes through glutamate transporter 1 (GLT1) and converted to glutamine for recycle to neurons and the re-generation of glutamate. Neurons can transfer damaged axonal mitochondria to astrocytes for autophagic degradation (mitophagy) whereas astrocytes can transfer healthy mitochondria to adjacent neurons as a neuroprotective and neurorecovery mechanism after stroke.

The diverse ability to perform OXPHOS and glycolysis between neurons and astrocytes is proposed to be related to inter-cellular coupling of glucose metabolism by the astrocyte-neuron lactate shuttle (Pellerin and Magistretti, 1994; Díaz-García et al., 2017; Figure 2). Glutamate released from active neurons activates astrocytic glycolysis and the production of lactate, which is used by neurons for synaptic activities (Mächler et al., 2016). Astrocytes take up more glucose than their energetic needs, which suggests their role in maintaining an extracellular lactate pool for neuronal use (Chuquet et al., 2010). In Drosophila, glial cells express glycolytic enzymes to produce lactate and alanine, which are then used to derive pyruvate for neuronal OXPHOS (Brooks et al., 1999; Volkenhoff et al., 2015). Lactate, pyruvate, and ketone bodies cross cell membranes through proton-linked monocarboxylate transporters (MCTs) (Halestrap, 2011; Figure 2). Knocking down astrocytic lactate transporter MCT4 in mouse hippocampus leads to memory retention, supporting the role of astrocytic lactate for long-term synaptic plasticity and memory (Suzuki et al., 2011). A recent *in vivo* study using a genetically encoded FRET sensor *Laconic* revealed a lactate gradient from astrocytes to neurons (Mächler et al., 2016). Astrocyte-to-neuron lactate shuttle was also found to be more functional in young hippocampi whereas aged neurons exhibit reduced dependence on astrocytic lactate with disrupted metabolic crosstalk (Drulis-Fajdasz et al., 2018). Perturbations to neuron-astrocyte metabolic coupling are seen in multiple neurodegenerative diseases (Rama Rao and Kielian, 2015). Decreased expression of astrocytic MCTs and defective neuron-astrocyte coupling of glucose metabolism was found in mouse models of AD and amyotrophic lateral sclerosis (ALS) (Ferraiuolo et al., 2011; Ding et al., 2013). A decline in levels of lactate, glucose, and other glycolytic intermediates was seen in the cerebrospinal fluid of Parkinson’s patients (Ohman and Forsgren, 2015).

### Neuron-Astrocyte Coordination of Fatty Acid Metabolism

Another metabolic coupling between neurons and astrocytes that was described recently is the coordination of fatty acid metabolism (Liu et al., 2015, 2017; Rambold et al., 2015; Ioannou et al., 2019; Figure 2). Lipids constitute 50% of the brain dry weight, mainly as fundamental component of membrane structures (Bruce et al., 2017). Since neurons are known to have minimum capacity to catabolize fatty acids (Schonfeld and Reiser, 2017), unused or recycled neuronal lipids are transported to astrocytes and stored in lipid droplets (LDs) before the metabolism in mitochondria (Liu et al., 2017; Ioannou et al., 2019). Sequestering of excessive fatty acids in LD could prevent lipotoxicity and mitochondrial dysfunction (Listenberger et al., 2003). In Drosophila, LD formation in niche glia under oxidative stress dampens intracellular ROS generation and oxidation of polyunsaturated fatty acids, which protects glia and neuroblasts from the peroxidation chain (Bailey et al., 2015). Upon nutrient depletion, LDs can deliver FAs to mitochondria for β-oxidation (Greenberg et al., 2011; Khor et al., 2013), and LD accumulation was found in olfactory bulb and vestibular nucleus of mice with dysfunctional mitochondria (Liu et al., 2015). Notably, apolipoprotein E-ε4 (APOE4), the strongest genetic risk factor for AD (Corder et al., 1993), reduces neuron-to-astrocyte transfer of fatty acids, which could be the underlying mechanism of the lipid dyshomeostasis seen in the disease (Liu et al., 2017). Additionally, LDs were also found to buildup in microglia of aged mouse or human brains, which then trigger ROS production and secretion of pro-inflammatory cytokines, and contribute to neurodegeneration (Marschallinger et al., 2019).

### Cholesterol Metabolism Across Neuron and Astrocyte

Brain contains ~20% cholesterol of the body (Zhang and Liu, 2015), which is essential for membrane fluidity, vesicle formation, and synaptic transmission (Björkhem and Meaney, 2004). Brain synthesizes its own cholesterol due to the block of cholesterol-carrying lipoproteins across the BBB. Since neurons acquire extra cholesterol for synaptogenesis from astrocytes (Mauch, 2001), and brain cholesterol needs to be hydroxylated to 24-hydroxycholesterol in neurons before excretion (Bjorkhem et al., 1998; Meaney et al., 2002), cholesterol transport from astrocytes to neurons is vital for synaptic function and sterol homeostasis in the brain. Upon co-culture, astrocytes stimulate neuronal neurite outgrowth, which is impaired when astrocytes without SREBP2 (a cholesterol synthesis regulatory protein) are used. *In vivo*, SREBP2 knockout in astrocytes alters brain development and impairs behavioral and motor functions in mice (Ferris et al., 2017), suggesting astrocyte-to-neuron cholesterol trafficking is key for brain development and synaptic function. APOE is the primary apolipoprotein that transports cholesterol in the brain. APOE lipoproteins carrying cholesterol and phospholipids are effluxed of astrocytes and uptaken into neurons by binding to lipoprotein receptors (Pfrieger and Ungerer, 2011; Figure 2). APOE-deficient mice exhibit markedly reduced cholesterol levels in hippocampus, and AD-like behavioral and synaptic impairments (Champagne et al., 2002; Levi et al., 2005). Astrocytes and neurons expressing APOE4 have reduced capacity in secreting or binding to cholesterol and phospholipids (Mahley, 2016). LDL receptor and LRP1 from LDL receptor family are main receptors to uptake APOE-containing lipoprotein particles to neurons. LRP1 knockout in forebrain neurons disrupts lipid metabolism, and leads to neuroinflammation and synapse loss (Liu et al., 2010). During aging, mRNA levels of the rate-limiting enzyme of cholesterol synthesis HMGCR decrease in astrocytes (Boisvert et al., 2018), which could contribute to dendrite atrophy and synaptic dysfunction seen in the aging brain. A study in AD-patient iPSC-derived neurons suggests that neuron-specific activation of cholesterol degradation could be a potential therapeutic target to alle*via*te Aβ and Tau pathology in AD (Van Der Kant et al., 2019).

### Additional Mechanisms of Neuron-Astrocyte Metabolic Interactions

Other than the coupling of glucose and lipid metabolism, additional metabolic interactions between neurons and astrocytes are essential for brain functions. Neuronal levels of reduced glutathione (GSH), the most abundant redox modulator eliminating ROS in the brain, are found to be synthesized using precursors amino acids, including glycine and cysteine, from astrocytes. GSH released from astrocytes is cleaved by γ-glutamyl transpeptidase on astrocyte surface to glycine and cysteine before being uptaken by neurons for GSH synthesis (Dringen, 2000). Inhibition of GSH biosynthesis in astrocytes triggers neuronal toxicity, and depletion of GSH during aging leads to microglia activation and increased neuronal susceptibility to cell death (Hirrlinger et al., 2002; Lee et al., 2010).

Intra-cellularly, damaged mitochondria are removed *via* mitophagy. Inter-cellularly, neurons release damaged axonal mitochondria to adjacent astrocytes for autophagic degradation (Chung-Ha et al., 2014). Conversely, astrocytes can transfer healthy mitochondria to adjoining neurons for ATP production and recovery from stroke (Hayakawa et al., 2016; Figure 2). When extracellular mitochondria are removed from astrocyte-conditional media, their neuroprotective effect is abolished (Hayakawa et al., 2016). At the molecular level, astrocyte-generated ATP, together with its degradation product adenosine, is involved in supporting synaptic transmission and neuronal excitability (Chen et al., 2013; Tan et al., 2017).

## Perspective

The high energy demand of the brain renders it sensitive to changes in bioenergetic capacity. Disruptions to substrate availability and/or mitochondrial function are well-known hallmarks of brain aging and a variety of age-associated neurodegenerative disorders. Distinguishing cell type-specific bioenergetic contributions to brain aging is particularly of significance because of previous findings linking aging with diverse metabolic changes between different brain cells. Elucidating the cellular specificity and inter-cellular coupling within the brain bioenergetic system is also critical to understand the mechanisms underlying selective cellular vulnerability in neurodegeneration. Mitochondrial enhancers targeting all brain cells, such as creatine, coenzyme-Q, and Mito-Q, have thus far exhibited limited efficacy for neurodegenerative diseases (Moreira et al., 2010; Chaturvedi and Flint Beal, 2013; Murphy and Hartley, 2018). While such a lack of success may be ascribed to multiple reasons including window of intervention and translatability of existing animal models (Murphy and Hartley, 2018), for neurodegenerative diseases that typically harbor complex and dynamic etiologies, precision strategies targeting specific cells or inter-cellular metabolic interactions may be needed at different disease stages. For stages when reactive astrocytes and microglia are playing a central role, interventions that facilitate their metabolic reprogramming could be more effective than direct OXPHOS enhancers (Baik et al., 2019; Polyzos et al., 2019). Recent methodological advances have started to enable targeted delivery of therapeutics to different brain cells *via* nanoparticles or viral carriers (Zhang et al., 2016; Sharma et al., 2018). Furthermore, emerging intra- and inter-cellular strategies targeting non-energetic facets of the mitochondria have manifested encouraging efficacy (Fang et al., 2019; Joshi et al., 2019), which suggest an necessity of combinational therapies for these currently incurable diseases.

## Author Contributions

All authors contributed to the discussion of content, generation of figures, and writing, review, editing of the manuscript.

### Conflict of Interest

The authors declare that the research was conducted in the absence of any commercial or financial relationships that could be construed as a potential conflict of interest.
